# Sub-THz and THz Cherenkov radiation source with two-dimensional periodic surface lattice and multistage depressed collector

**DOI:** 10.1038/s41598-024-74374-9

**Published:** 2024-10-13

**Authors:** Amy J. MacLachlan, Liang Zhang, Ivan V. Konoplev, Alan D. R. Phelps, Craig W. Robertson, Philip MacInnes, Colin G. Whyte, Kevin Ronald, Adrian W. Cross, Mark A. Henderson

**Affiliations:** 1 Department of Physics, SUPA, University of Strathclyde, Glasgow, G4 0NG UK; 2 Culham Centre for Fusion Energy, UK Atomic Energy Authority, UKAEA, Culham Science Centre, Abingdon, OX143DB UK

**Keywords:** Physics, Applied physics, Electronics, photonics and device physics

## Abstract

We present the theory, concept and design of an efficient, megawatt coherent Cherenkov radiation source based on a two-dimensional periodic surface lattice (2D-PSL) cavity combined with a novel energy recovery system for the generation of highly efficient (> 50%) single-frequency radiation. We demonstrate the scalability of the transverse dimension of the 2D-PSL cavity of the Cherenkov source and thus the potential for efficient, continuous-wave, high-power (> 1 MW) operation; fundamental to the eventual realization of clean, fusion energy. These new sources, with the capacity to operate in the 0.1-10THz range, hold strong promise to address the long-standing “Terahertz gap”. By combining a Cherenkov oscillator driven by a non-gyrating beam with an innovative four-stage depressed collector energy recovery system, the overall device efficiency can be increased to be competitive with gyrotrons in the requirements for heating and current drive in fusion plasma. In these Cherenkov devices, the frequency independence of the magnetic guide field enables advantageous frequency scaling without deployment constraints, making them especially attractive for high-impact applications in fusion science, turbulence diagnostics, non-destructive testing and biochemical spectroscopy. The novel energy recovery techniques presented in this paper have broad applicability to many electron-beam driven devices, bringing revolutionary potential to future THz source technologies.

## Introduction

The theory and concept of powerful, sub-terahertz and terahertz (THz) sources based on a Cherenkov interaction mediated by two-dimensional periodic surface lattice (2D-PSL)^1–4^ cavities has been developed and discussed. Theoretical and numerical studies show that the overall efficiency of these radiation sources^5^ can be significantly improved if combined with a multi-stage depressed collector^6–8^, potentially outperforming alternative techniques for generating powerful millimeter and sub-millimeter EM radiation. Ground-breaking scientific and industrial applications of these new sub-THz (below 1THz) and THz (1-10THz) sources include: plasma physics and plasma scattering diagnostics, bioscience and chemistry (high-frequency electron paramagnetic resonance (EPR), nuclear magnetic resonance (NMR) and dynamic nuclear polarization (DNP) spectroscopic techniques), advanced radar systems, remote sensing (space debris; dust cloud formation; environmental monitoring) communications and non-destructive testing of materials^9–13^.

To enable CW ~1 MW operation, the transverse dimension of the interaction region (cavity) *D*, must be significantly larger than the source wavelength $\lambda,$ i.e. oversized parameter, ${o}_{p}=D/\lambda>9$. This ensures that the EM power density within the interaction region is sufficiently low so as to avoid overheating and RF breakdown. In highly oversized interaction cavities, hundreds to thousands of EM eigenmodes can be excited by the electron beam. The conditions imposed on the EM field structure by the 2D corrugation can enable the excitation of a single eigenmode, demonstrating single-mode excitation and steady-state operation (at a given frequency). Suitable parameters for the 2D-PSL geometry, electron beam, and magnetic guide field are estimated^14,15^ from analytical theory and optimized via numerical studies.

The Cherenkov interaction between a non-gyrating electron beam and the EM field is facilitated by the hybrid EH (i.e. having both *E*_z_ and *H*_z_ components) field structure of the 2D-PSL eigenmode, similar to that observed in a dielectric-lined waveguide. The eigenmode of the 2D-PSL cavity can be defined as a superposition of high radial order (large number of radial variations) near cut-off partial volume waves and high azimuthal order (large number of azimuthal variations) partial surface waves, resonantly coupled by the corrugated boundary^1,14^. The resonant coupling of the partial volume and surface waves, and resultant cavity EH eigenmode mediate a unique interaction between the EM waves and the non-gyrating electron beam, enabling coherent radiation output. The fundamental mode coupling principles discussed in this paper are also relevant to solid state physics, photonics and optics, where surface plasmon polaritons excited on periodic gratings can couple with plasmons^16^.

**Fig. 1:**
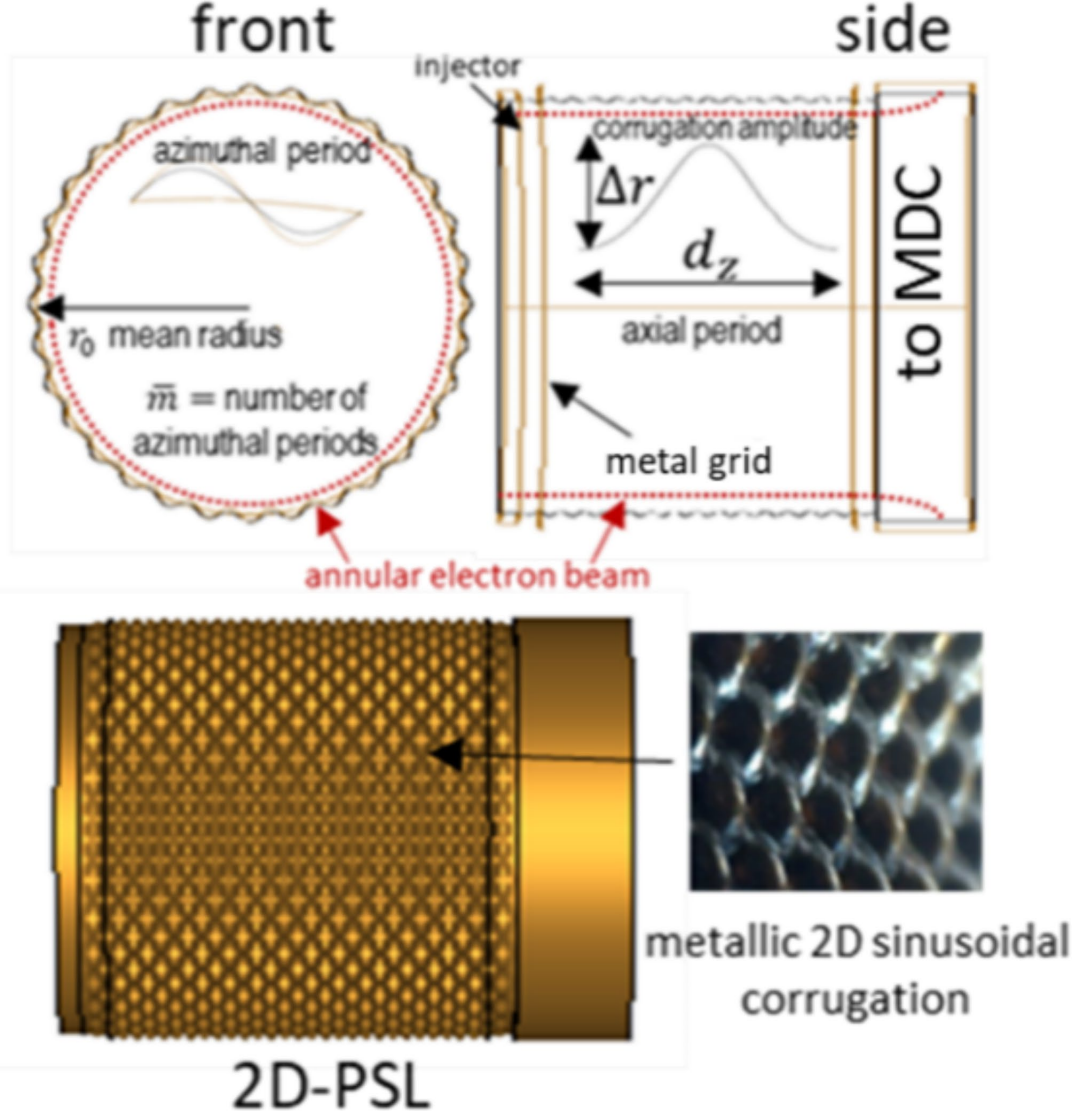
Conceptual diagram of oversized 2D-PSL oscillator illustrating the key parameters of the novel interaction structure.

Demonstrating high-order mode coupling in Cherenkov oscillators based on progressively oversized, ${o}_{p}\cong {9,12}$ 2D-PSL interaction structures would confirm the potential for long-pulse (quasi-CW) and CW sub-THz and THz sources offering from kilowatts to megawatts of output power. Very oversized $({o}_{p}\cong 12)$ sources operating just below the lower THz threshold, have the capacity to offer up to a megawatt of CW power in the 83–86 GHz range. These power capabilities, coupled with projected system efficiencies of >50% (with the inclusion of an energy recovery system) are well-aligned with the requirements for magnetically confined fusion initiatives. The parameters presented in this paper have been chosen to illustrate this potential. A conceptual diagram, describing the overall geometry and key parameters is presented in Fig. 1.

Here, we explore this potential by investigating and optimizing the nonlinear dynamics of the system to maximize the energy extraction, ensuring the radiation generated from different parts of the large diameter electron beam is coherently synchronized, and implementing a scheme for spent beam energy recovery^8^. The potential for energy recovery in gyrotrons (around 55% overall efficiency) has been investigated and improved over many years^17–22^. By considering an interaction with a non-gyrating electron beam^1–5^, spent beam electron energy recovery^8,23–27^ (with the potential to achieve > 55% efficiency) can be explored. In conventional Cherenkov devices (backward-wave oscillators or travelling wave tubes), used for satellite communication, the total wall-plug efficiency is demonstrated to be ~80%^6,7,23,24^. It seems reasonable that 2D-PSL sources could aspire to similar efficiencies by employing a multi-stage depressed collector energy recovery system^8^. Though multi-stage depressed collectors (MDCs) have been widely used in low-power traveling wave tubes (satellite communication), and have previously been designed for gyro-backward wave devices^25–27^, no studies into this method of energy recovery have hitherto been carried out for high-power, ${o}_{p}>9$ Cherenkov oscillators driven by thin, annular electron beams. We combine, for the first time, these contemporary research advances to investigate and demonstrate, via numerical modeling, an integrated overmoded Cherenkov oscillator with a multi-stage depressed collector^5,8^. We predict the potential to achieve competitive efficiencies at the frequency ranges relevant for applications in fusion and other high-impact areas.

## Results

### Dispersion analysis

The electromagnetic field structure inside an oversized cylindrical cavity with a two-dimensional sinusoidal surface corrugation (as defined by ${p=r}_{0}+\Delta{r} cos\left({\bar{m}}\phi\right){\cos}\left({\bar{k}}_{z}z\right)$ where ${\bar{m}}$ is the number of azimuthal periods and ${\bar{k}}_{z}$ is the lattice wavevector) is described by considering an equivalent smooth waveguide of finite length excited by a fictitious magnetic surface current, ${\varvec{j}}_{m}$ for which the following boundary condition applies:

1 $${\varvec{j}}_{m}=\varvec{n}\times\left[\nabla\left(p\varvec{E}\cdot\varvec{n}\right)\right]+i\omega{p}\varvec{n}\times\left[\varvec{n}\times\varvec{H}\right]$$

**Fig. 2:**
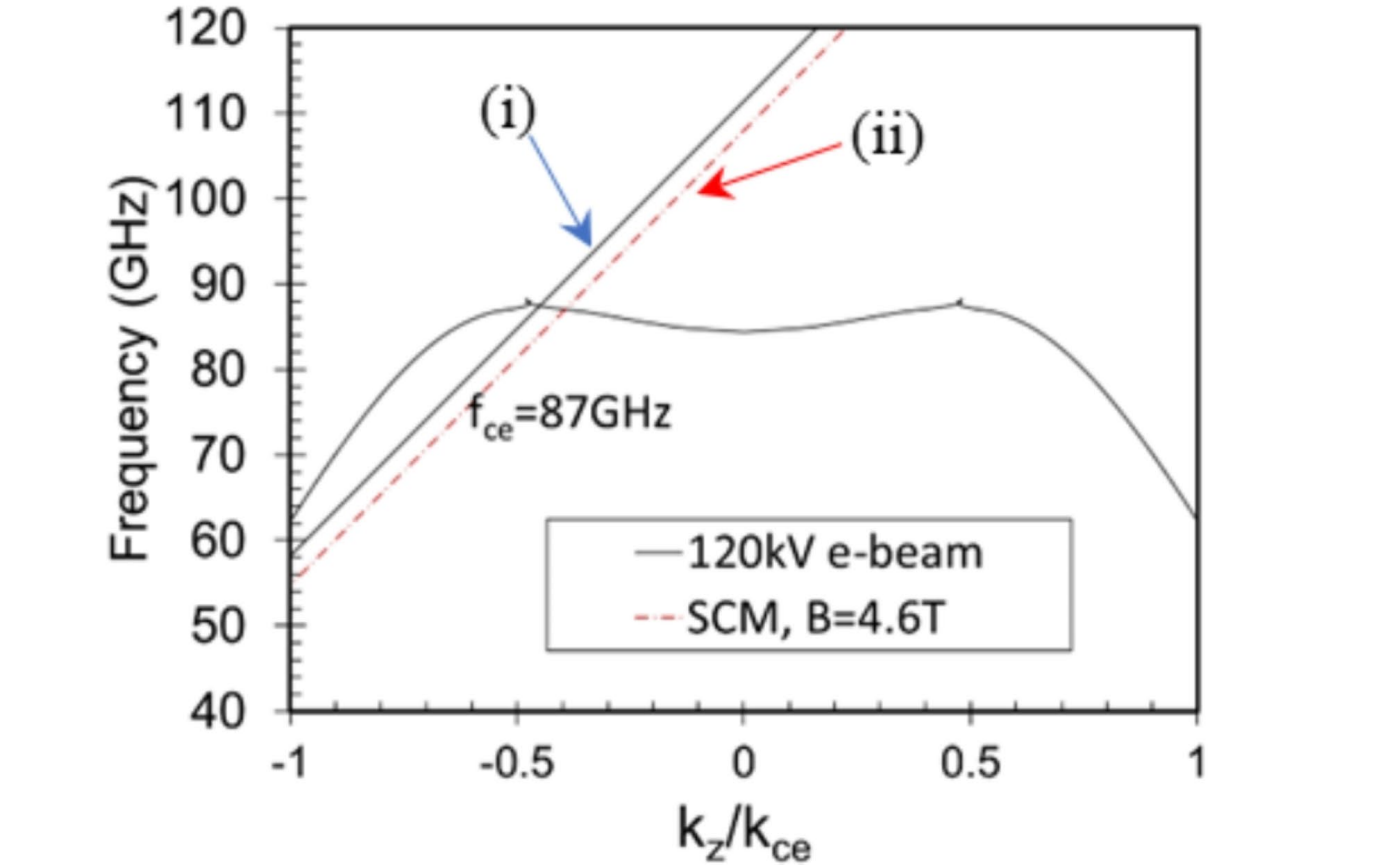
Illustration of the synchronism conditions showing crossing of the analytical dispersion and electron beam line (non-gyrating electrons, solid line) and electron cyclotron beam line (gyrating electrons, dashed line). The axial wavenumber${k}_{z}$ is normalized by the electron cyclotron wavenumber,${k}_{ce}$. The points of intersection illustrate (i) the backward wave interaction with the 120 keV electron beam at the operating frequency *f* ≅ 87 GHz and (ii) the frequency where the strongest cyclotron absorption (*f*_ce_ = 87 GHz) can be expected for B = 4.6T.

Vector quantities (including the normal unit vector, ***n***) are denoted in bold. The transverse electric ***E*** and magnetic ***H*** fields are expanded as slow wave packets of varying amplitudes filled with oscillating terms. The electric and magnetic fields are described as a summation of the complete set of eigenmodes^3,28^. Integrating the Poynting vector over the cylindrical cross section gives the power transmitted through the waveguide. For the stationary regime, where localized surface fields are coupled with a non-propagating (near cut-off) azimuthally symmetric TM_0,N_ volume field, the wave equation is defined:

2 $${\nabla}_{z}^{2}{C}_{q}^{v,s}\left(z\right)+\omega\delta{C}_{q}^{v,s}\left(z\right)\mp\frac{{\bar{\omega}}\Delta}{{c}^{2}}{C}_{q}^{v,s}\left(z\right)={N}_{v,s}\oint{\varvec{j}}_{m}\cdot{\varvec{H}}_{q}^{*}d\sigma$$

where ${C}_{q}^{v,s}$ are the slowly varying amplitudes of the volume “*v*” and surface “*s*” fields, *q* is the eigenmode number, $\delta$ is the Bragg detuning (which can be complex with the imaginary component describing losses) and ${\bar{\omega}}$ is the mean frequency of the volume and surface fields. The wave norm ${N}_{v,s}$ for the volume (−) and surface fields (+) is ${N}_{v,s}=i\omega{\epsilon}_{0}/\oint{\varvec{H}}_{q}.{\varvec{H}}_{q}^{*}d\sigma$, where ${\varvec{H}}_{q}^{*}$is the complex conjugate of the magnetic field of the eigenmode. Equation (1) is substituted into the right-hand side of Eq. (2) which (for a non-trivial solution) must be non-zero. Averaging over the fast oscillation terms and renormalizing as outlined in^28,29^ gives the coupled 2D-PSL dispersion relation:

3 $$\left({\omega}_{e}^{2}-{{\Lambda}}^{2}\right)\left\{{{\Lambda}}^{4}-2{{\Lambda}}^{2}\left[\left(2+{{\Gamma}}^{2}+{\omega}_{e}^{2}\right)+{\left(2-{{\Gamma}}^{2}+{\omega}_{e}^{2}\right)}^{2}\right]\right\}=2{\alpha}^{4}\left(2-{{\Gamma}}^{2}+{\omega}_{e}^{2}-{{\Lambda}}^{2}\right)$$

where ${\Lambda}$ is the normalized wave vector, ${\omega}_{e}$is a variable angular frequency, ${\omega}_{c}^{v,s}$ is the angular cut-off frequency of the volume and surface modes, $\Delta\omega=\sqrt{{(\left({\omega}_{c}^{v}\right)}^{2}+{\left({\omega}_{c}^{s}\right)}^{2})/2}$, ${\Gamma}=c{\bar{k}}_{z}/\Delta\omega$ is a geometrical detuning parameter, and $\alpha$ is the normalized coupling coefficient (defining the strength of the volume and surface field coupling). The coupling coefficient is expressed:

4 $$\alpha=\frac{\pi{\omega}^{2}{\epsilon}_{0}{r}_{0}{\Delta}r}{2}\sqrt{\frac{\left({H}_{q,\tau}^{*,v}\cdot{H}_{q,\tau}^{s}\right)\left({H}_{q,\tau}^{*,s}\cdot{H}_{q,\tau}^{v}\right)}{\oint\left({H}_{q}^{*,v}\cdot{H}_{q}^{v}\right)d\sigma\oint\left({H}_{q}^{*,s}\cdot{H}_{q}^{s}\right)d\sigma}}$$

where ${\epsilon}_{0}$ is the permittivity of free space, ${H}_{q,\tau}^{v,s}$ is the tangential magnetic field of the ‘partial’ volume and surface fields of the eigenmode and ${H}_{q,\tau}^{*,v,s}$is the complex conjugate of the tangential volume and surface fields. The coupling coefficient has been estimated from previous studies^15,28^ and by comparing the analytical dispersion plots with the numerical Particle-in-Cell (PiC) simulations. Solving Eq. (3) for ${r}_{0}=15.3$ mm, ${d}_{z}=1.58$ mm, $\alpha=0.7$ gives the dispersion of the coupled cavity eigenfield, presented in Fig. 2.

The 2D PSL synchronism condition (black line in Fig. 2) is defined: $$\omega={(k}_{z}+l{\bar{k}}_{z}){v}_{z}\pm{\omega}_{p}$$, where $${\omega}_{p}$$ is the plasma frequency of the electron beam and $${\bar{k}}_{z}=2\pi/{d}_{z}$$ is the axial wavenumber of the 2D-PSL. In accordance with Floquet’s Theorem, the dispersion curve is periodic along $${k}_{z}$$ with $$l{\bar{k}}_{z}$$ describing the spatial harmonic ($$l=1).$$ Fig. 2 shows the predicted backward wave interaction between a 120 keV electron beam (black line) and the 2D-PSL eigenfield. Similarities with the dispersive characteristics of degenerate band edge modes^30,31^ are observed. To maximize the interaction efficiency, it is important to avoid the region of the electron cyclotron absorption resonance by carefully considering the magnitude of the axial magnetic field. The synchronism condition of the cyclotron mode and the electron beam (Fig. 2, dashed line) can be written: $${\omega}_{ce}={k}_{z}{v}_{z}+{{\tilde{n}}({\Omega}}_{ce})$$ where $${\tilde{n}}$$ is the electron cyclotron harmonic ($${\tilde{n}}=1$$ in the Fig. 2) and $${{\Omega}}_{ce}$$ is the angular electron cyclotron frequency:

5 $${{\Omega}}_{ce}=\frac{eB}{\gamma{m}_{e}}$$

Here, $e$ is the electronic charge, *B* is the magnitude of the magnetic guide field, ${m}_{e}$is the electron mass, $\gamma$is the relativistic Lorentz factor, $\gamma\sim1+{V}_{e}/511$, ${V}_{e}$ is the electron energy in keV of a single electron and 511 keV is the electron rest mass energy. Figure 2 shows a backward wave interaction between the cavity eigenfield and the ${\tilde{n}}=1,$ slow electron cyclotron mode at 87 GHz (corresponding to B = 4.6T) in the negative *k*_z_ region of the first Brillouin zone. The cyclotron absorption is predicted to occur when the magnitude of the axial magnetic field is $B\sim(4.0-4.6){\text{T}}$. In these devices, the optimum interaction efficiency is typically observed when the magnitude of the axial magnetic guide field is at least ~2T from the magnetic field corresponding to the electron cyclotron resonance. To drive the beam close to the corrugated wall, and avoid electron cyclotron absorption, an axial magnetic guide field of 6.5-6.7T has been chosen.

## Electrodynamic simulations

**Fig. 3:**
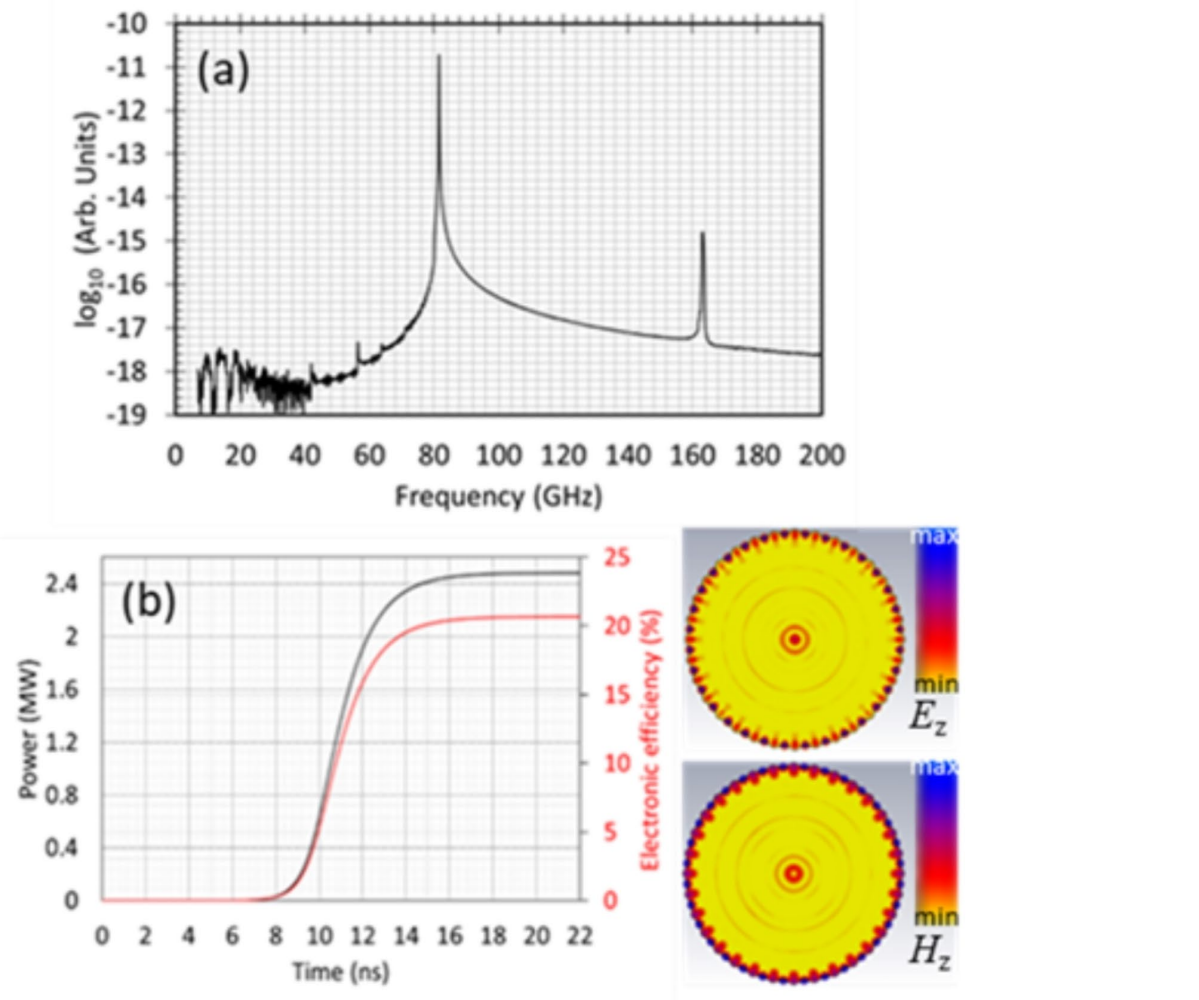
(**a**) Output radiation spectrum (logarithmic scale where the numbers on the axis represent 10^N^) showing output at 83 GHz and second harmonic 166 GHz and (**b**) Time dependence of the output power and interaction efficiency for the surface wave source based on the 2D-PSL cavity with $D/\lambda=12$. The inset shows the contour plots of the *E*_z_ (top) and H_z_ (bottom) eigenmode field components^35^.

The parameters of the 2D-PSL interaction cavity have been calculated from analytical theory^3,15,28^ and optimized by numerical modeling. The generation of radiation at 83–86 GHz has been simulated using oversized ${o}_{p}=D/\lambda\cong{9,12}$ 2D-PSLs with: mean radii, ${r}_{0}=15.3$ mm, 20 mm; number of azimuthal periods, ${\bar{m}}=26$, 32; axial periodicity, ${d}_{z}=2\pi/{\bar{k}}_{z}=1.58$ mm, 1.54 mm and corrugation amplitudes, $\Delta{r}$ = 0.52 mm, 0.60 mm. The total length of the cavities (comprised of $n=16$ periods and two additional tapered corrugation periods) is $L=18{d}_{z}$. In Fig. 3(a), the spectral peaks (plotted on a logarithmic scale) at 83 GHz (fundamental harmonic) and 166 GHz (second spatial harmonic) demonstrate excellent spectral purity. Figure 3(b) shows powerful (2.5 MW), efficient (~ 20%) steady state operation (Fig. 3b) facilitated by the formation of the complex, hybrid eigenmode (contour plots, right).While this power can be sustained over tens of nanoseconds (i.e. pulsed regime), for CW operation, the output power must be within the 0.5-1 MW range. The majority (> 95%) of the output power is in the high-order, HE_32,1_ eigenmode with a further 100 kW contributed by the sum of the azimuthally symmetric TM_0,N_ modes (where *N* = 1.10). Numerical studies of the 86-GHz, $D/\lambda=9$ 2D-PSL oscillator exhibit similar behavior to that shown in Fig. 3, with single frequency excitation and substantial (~2 MW) power in the HE_26,1_ coupled eigenmode.

**Fig. 4:**
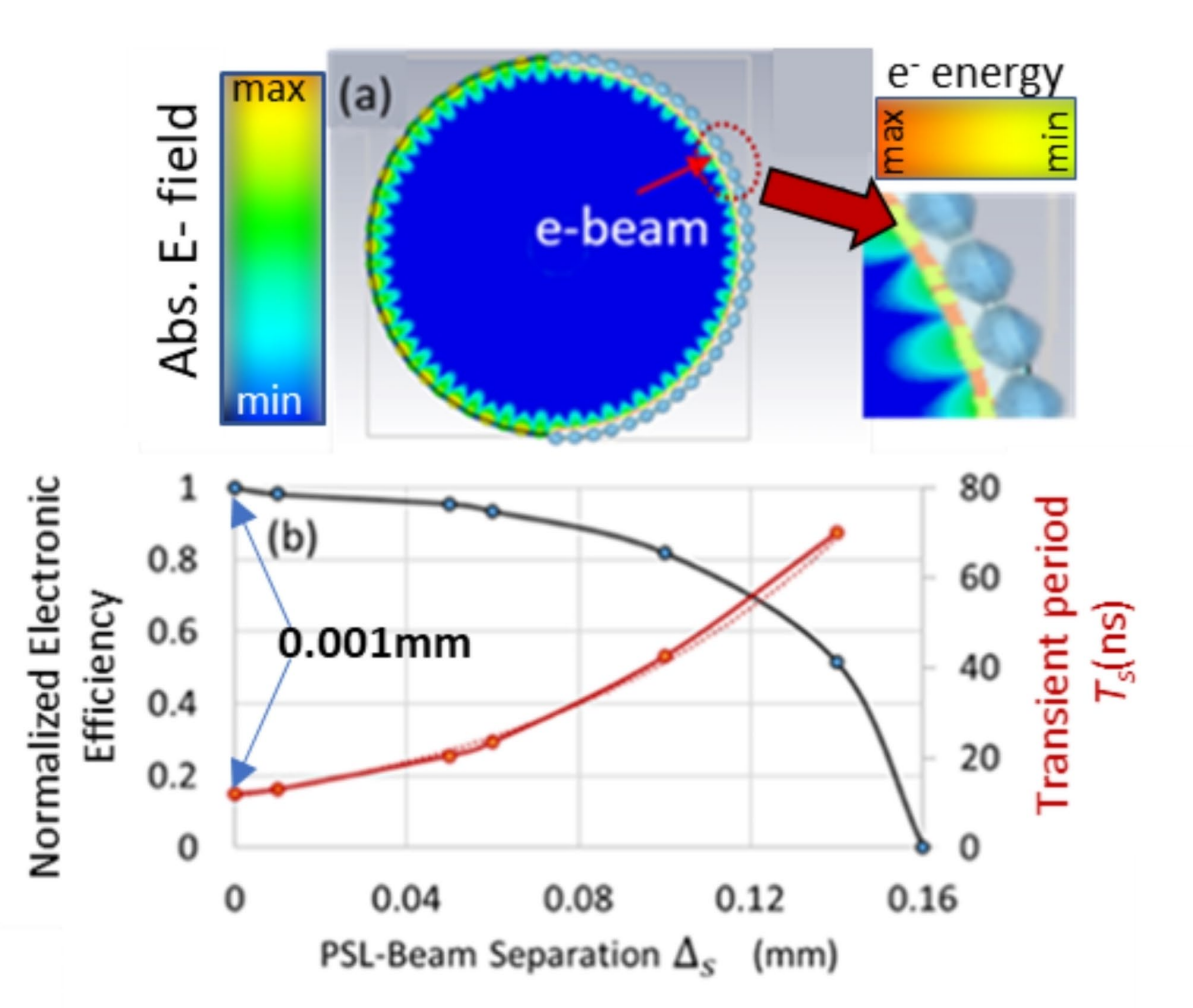
(**a**) A 2D contour plot showing the surface field of the 2D PSL extending beyond the 2D corrugation (colored gray) where it is overlapped by the electron beam (orange/yellow annulus). The inset shows the close-up view of the edge of the 2D-PSL cavity. The orange and yellow coloring of the beam (see inset colorbar) shows the relative variation in the electron energies. The contour plot of the *E*_z_ field is shown by the colorbar on the right. (**b**) The dependences of the normalized electronic efficiency (left axis) and transient/saturation period ${T}_{s}$ (right axis) on the electron beam-grating separation ${\Delta}_{s}$ measured from the peak of the corrugation to the outer radius of the electron beam. The smallest value of ${\Delta}_{s}$ is 0.001 mm .

To observe an efficient interaction between the electron beam and the cavity eigenmode, the beam trajectories should overlap with the high-intensity *E*_z_ field (Fig. 4(a) and inset to Fig. 4(a)). The yellow-orange ring in Fig. 4(a) represents the varying electron energies of the annular beam, positioned close (${\Delta}_{s}=0.01$ mm) to the 2D-corrugation (shown as the grey outer ring). As the distance (${\Delta}_{s}$) between the outer radius of the beam and the minimum radius of the corrugation (Fig. 4a) is increased from ${\Delta}_{s}=0.01$ mm to ${\Delta}_{s}=0.05$ mm, the electron beam moves further from the maximum of the *E*_z_ field, and the efficiency drops to 19.8%. When ${\Delta}_{s}=0.1$ mm, the efficiency drops to 17% and the time taken for the wave-beam interaction to reach steady-state increases. Figure 4(b) shows the efficiency and transient/saturation time ${T}_{s}$ as a function of the distance, ${\Delta}_{s}$. When the separation between the beam and the corrugated wall is increased to ${\Delta}_{s}=0.16$ mm, no interaction is observed within the 100ns time duration, due to insufficient wave-beam coupling. Studies investigating the coupling between metal periodic corrugated structures and electron beams were carried out in^32,33^ and the exponential decay of this coupling was experimentally observed^34^. While the formation of thin annular beams is outside the scope of this paper, such beams can be formed using magnetic field compression at the cathode i.e. from ~1 mm to ~ 0.14 mm. Simulations confirm that the strong, uniform magnetic guide field allows the beam to be transported through the structure without electrons being lost to the wall.

## Multi-stage depressed collector

**Fig. 5:**
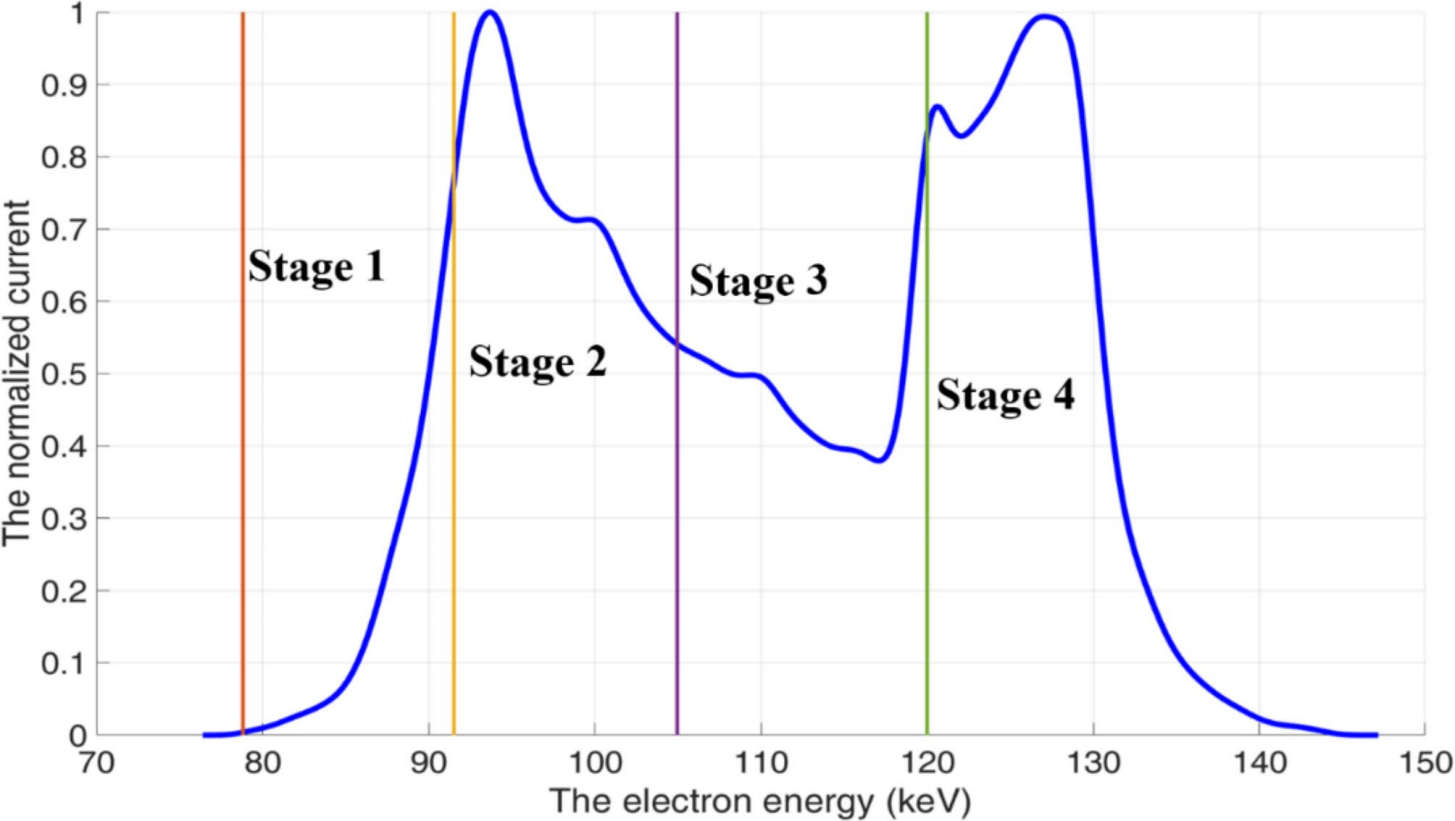
The current normalized to the peak current as a function of the electron energy. The vertical lines (orange, yellow, purple, green) illustrate the optimal potentials of the 4-stage depressed collector.

**Fig. 6:**
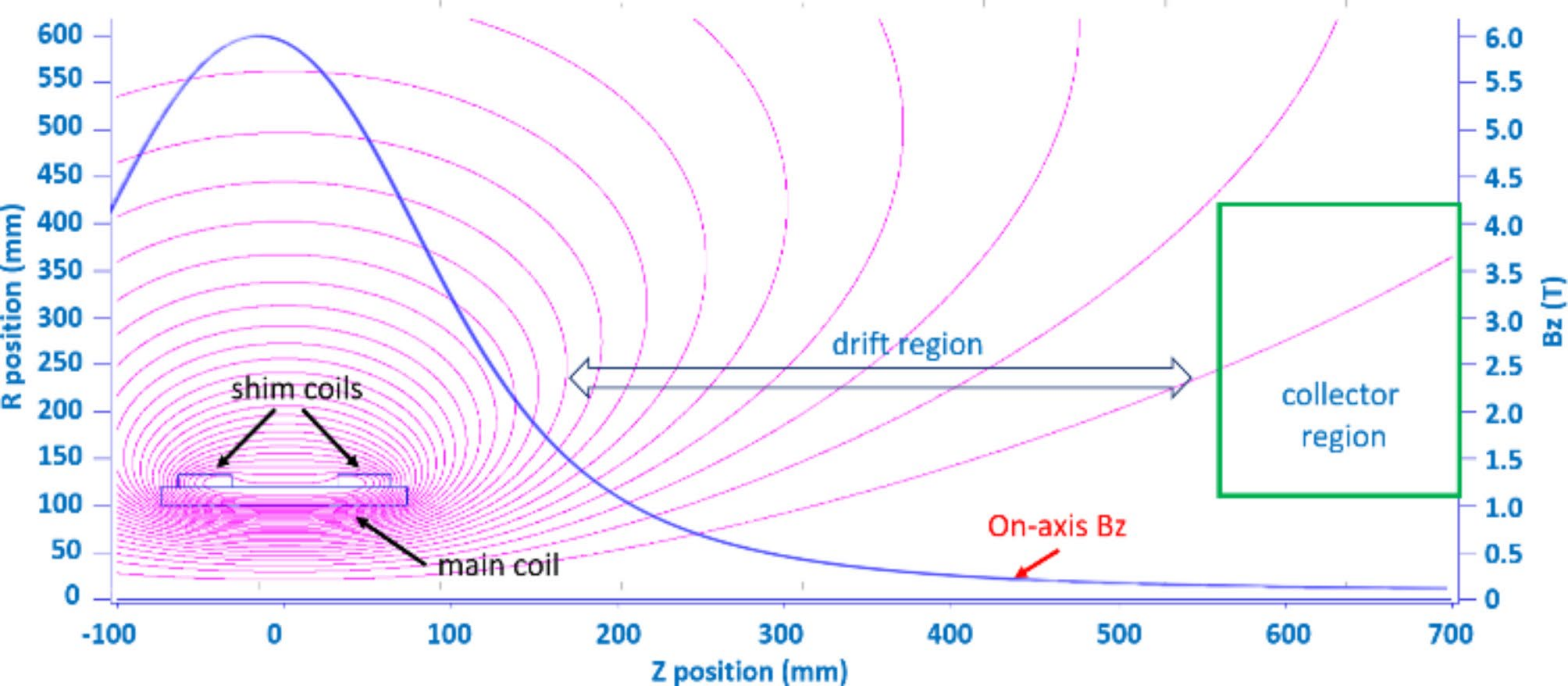
Configuration of superconducting magnet and the magnetic field profile used for the simulations.

**Fig. 7:**
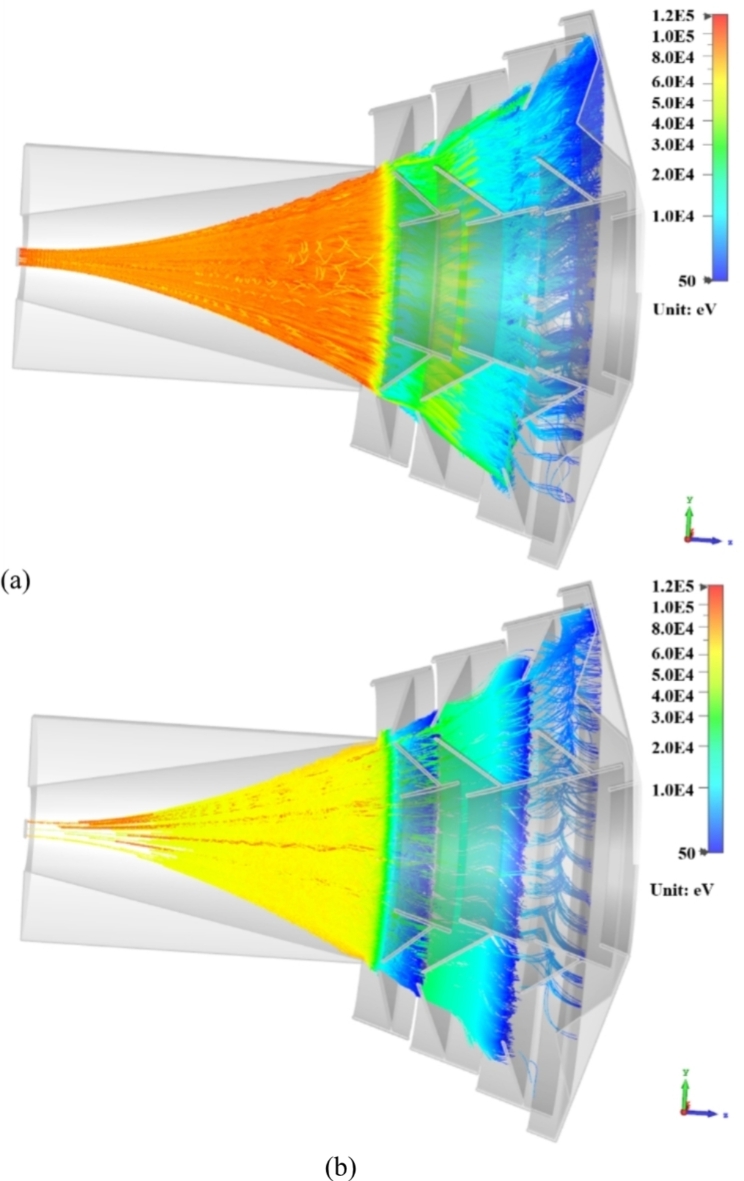
Plot of particle trajectories showing (**a**) the beam (primary) electron trajectories and (**b**) the trajectories of the secondary electrons. The secondary electrons are emitted from the collisions of the beam electrons with the electrodes of the 4-stage MDC.

Once the wave-beam interaction has reached the steady-state, significant (up to 80%) energy remains in the spent electrons. Recovering energy from the spent beam reduces the thermal load of the beam dump area, and increases the overall efficiency of the Cherenkov oscillator. Multi-stage depressed collectors (MDC)^6–8,25–27^ produce a retarding electrostatic field, forcing the spent electrons to lose their kinetic energy as they pass through the collection region. The spent electrons are collected by the MDC electrodes with reduced kinetic energy and a current loop is created to ‘recover’ the energy from the spent beam. The power collected can be written:

6 $${P}_{col}=\sum_{M}{V}_{M}{I}_{M}$$

where *M* is the number of collection stages, ${V}_{M}$ is the electric potential of the stage *M* and ${I}_{M}$ is the collected current on the *M*’th stage of the MDC. The combined efficiency of the oscillator and depressed collector, ${\eta}_{tot}$ is calculated from^26^

7 $${\eta}_{tot}=\frac{{P}_{i}}{{P}_{b}-{P}_{col}}=\frac{{\epsilon}_{out}{\eta}_{e}}{1-{\eta}_{col}(1-{\eta}_{e})}$$

where ${\epsilon}_{out}$ is the efficiency of the output coupler (i.e. the ratio of the output power to the power generated in the interaction cavity), ${\eta}_{col}={P}_{col}/{(P}_{b}-{P}_{i}$) is the collection efficiency, ${P}_{b}$ is the power in the electron beam and ${P}_{i}$ is the power generated in the wave-beam interaction. The collection efficiency largely depends on the properties of the spent beam. Figure 5 illustrates the current of the spent beam (normalized to the peak current) as a function of the electron energy and the optimum potentials at each stage of the 4-stage depressed collector, while Table 1 shows the theoretical collection efficiency for collectors with 1–5 stages. In practice, a compromise between the complexity of the collector design and the efficiency of the energy recovery should be sought. For many applications, the small uplift in efficiency (Table 1) gained by adding fourth (up to 2.6%) and fifth (up to 1.6%) collection stages is outweighed by the associated difficulties in the design and implementation of the power supply. The 2D-PSL Cherenkov oscillator is driven by an annular electron beam (rather than a pencil beam as used in conventional Cherenkov devices) immersed in a strong magnetic guide field. While the axial magnetic field inside the 2D PSL interaction region, must be sufficiently strong to guide the beam with minimal perpendicular velocity, the B-field in the MDC region should be gradually reduced to allow the electrons of varying energy to spread out before entering the collector. This, over the different stages of the MDC, is similar in principle to an energy spectrometer. Figure 6 shows the configuration of the superconducting coils and the magnetic field inside the interaction region and depressed collector, which were designed and simulated using SuperFish^36^. Figure 6 indicates that the magnetic field remains relatively strong over a large distance. The drift region, required to increase the electron energy spread of the thin annular beam before it enters the collector, is annotated in Fig. 6. The position of the coils and the driving current density are listed in Table 2. Although additional coils with reverse drive current can be used to increase the decay of the magnetic field in this region, the resultant increase in *B*_r_, and growth in radial momentum, reduces the collection efficiency. A realistic emission model was applied to investigate the impact of secondary electron emission on the amplitude of the backstreaming current and overall MDC efficiency. The inclusion of the secondary electrons increased the backstreaming current to 0.52 A. The trajectories of the primary and secondary electrons are shown in Fig. 7.

**Table 1:** Theoretical collection efficiency ${\eta}_{col}$ at stage number, *M*.

M	1	2	3	4	5
${\eta}_{col}$	66.4%	74.6%	92.1%	94.7%	96.3%

**Table 2:** Configuration of the coils in the superconducting magnet.

Coil no.	Inner radius (mm)	Outer radius (mm)	Initial axial position (mm)	Final axial position (mm)	Current density (A/mm^2^)
1	100.00	119.00	-73.70	73.70	363.6
2	120.00	133.00	-63.70	-31.80	363.6
3	120.00	133.00	31.80	63.70	363.6

## Discussion

The electrodynamic PiC simulations indicate that an axial magnetic guide field of ~3T is sufficient to prevent the electron beam from impinging on the metal corrugation. However, as predicted from the theory (Fig. 2)^15^, a magnetic field magnitude of 6.5-6.7T is required for optimum efficiency. To obtain the results presented in Fig. 3, a thin (${\Delta}_{b}=0.14$ mm) annular, 100 A electron beam with predominantly axial velocity was injected close to the corrugated wall. The electron beam has a modest pitch angle, ${{\alpha}_{p}=v}_{\perp}/{v}_{\parallel}=$ 0.01 to ensure a small enough Larmor radius ~ 1.5 μm. Further increasing the magnetic field up to 9T (close to the upper range of most superconducting magnets) was found to reduce electronic efficiency by around 1.3%.

Previous studies have shown that the 2D-PSL devices operate more efficiently in the high current regime^14^. Shorter structures, compatible with an *I*$\ge$80A electron beam, are therefore advantageous. Such structures have the additional benefit of being easier to manufacture. However, for continuous operation, relatively low current densities are needed, and thus a compromise between the length of the interaction structure and the electron beam current must be reached. It was observed that a significant drop in efficiency, due to longitudinal mode competition, occurs when *n* is increased beyond 20. For longer structures (*n* ≥ 20) the electron beam current *I* must be reduced by a factor of 2, i.e. below the starting current of the high order longitudinal modes, to mitigate oscillatory behavior. Further studies are required to achieve the same 20% interaction efficiency for currents much lower than 40 A.

A major challenge that can hinder the stable, operation of the device, justifying the need for large ${o}_{p}$, is thermal loading. Ohmic losses, which constitute the main thermal load in the interaction region and can impact the effectiveness of the corrugation^37^, will be significantly increased by excessive surface roughness, impacting the device efficiency. Excess conductive losses occur when the surface roughness is comparable to the skin depth of the wave in the copper. Numerical studies^14,38^ indicate that increasing the surface roughness can reduce the efficiency by around 6%, showing that surface imperfections associated with the manufacturing process can significantly reduce the performance of the device.

Compared to less oversized 2D-PSL structures^14,15^, the electromagnetic fields of the ${o}_{p}=D/\lambda\cong{9,12}$ cavities behave more like those of a planar (parallel plate) corrugated waveguide, in which the surface field is localized within the grooves and decays rapidly from the corrugated surface^10,39,40^. Nevertheless, the 2D electromagnetic field contour plot presented in Fig. 4(a) indicates that the localized surface field is still extending beyond the corrugated boundary where it can efficiently couple with the electron beam. These results suggest that the ${o}_{p}$ limit has not yet been reached, further confirming the potential for continuous-wave output.

One possible way in which the electron beam can be injected further from the wall, minimizing the risk of undesirable interception, whilst still enabling strong coupling with the cavity eigenmode and thus retaining the high efficiency, is through the excitation of a higher radial order surface mode. Exciting a surface mode with an increased radial index and the same number of azimuthal variations, allows the annular beam to couple with a field maximum located further from the cavity wall. At this stage, only interactions involving the fundamental partial surface wave have been considered, and studies of the electron beam coupling with high order (radial index > 1) partial surface modes^1^ will be the subject of further research.

## Methods

The oversized Cherenkov oscillators operating at 83–86 GHz were simulated using the PiC solver of CST Particle Studio (CST-PS). The diameter (*D* = 40 mm) of the more oversized ${o}_{p}\cong12$ interaction region is sufficiently large to enable sustained 0.5-1 MW output. A detailed description of this general model, in which the Ohmic loss of the copper is considered, is presented in^37^.

In these studies the properties of the spent beam were exported from the CST-PS simulation of the 2D PSL interaction region. The spent beam parameters such as electron energy, velocity, and spatial distribution were post-processed from the exported data and used to calculate the theoretical collection efficiency for each stage of an MDC. These estimated values serve as theoretical targets to optimize the number of electrodes in the MDC for a given spent beam distribution. To mitigate the electron beam back-streaming (reflection), the minimum potential of the MDC electrodes was set to equal the minimum electron energy, while the maximum potential was matched to the initial electron beam voltage which also simplifies the power supply. The electrode potentials at the first to fourth collection stages are − 78.8 kV, -91.5 kV, -105.0 kV and − 120.0 kV respectively.

CST Particle Studio was used to design and optimize the geometry of the depressed collector. The 3D magnetic field in the simulation region was mapped from the SuperFish calculation results. Each collector stage contains 3 free parameters. For the 4-stage depressed collector, there are a total of 12 free geometric parameters. The number of free parameters of the MDC significantly increases with each additional collection stage thus requiring significant computational time for each parameter set. A hybrid optimization routine (manual parameter sweeps combined by new parameter sets generated from an evolution algorithm) was therefore used to reach the optimal design in a reasonable timeframe^25–27^. While one of the optimization goals was to maximize the collection efficiency, another objective was to minimize the back-streaming of primary electrons (reflection), which can travel back into the interaction region, generating noise and parasitic oscillations. In practice, the collection efficiency from the numerical simulation is always lower than the theoretical value (Table 1) due to geometric imperfections and electron backstreaming^25–27^. However, the simulations exhibit a similar general trend, with the percentage increase in efficiency decreasing at each stage. The simulated optimum collection efficiencies for the MDC with 3 (83.1%) and 4 (88.6%) stages are compared to the theoretical predictions of 92.1% and 94.7% respectively.

The results show that a good balance between the amount of energy recovered relative to the system complexity, can be achieved using a four-stage depressed collector. Reducing the potential at the first stage by 10% could eliminate the backstreaming electrons, with minimal sacrifice (~ 0.7%) in collection efficiency. Although the back-streaming current is comprised mainly of reflected electrons, secondary electrons (emitted from the collector’s electrodes) are another contributory factor, detrimental to the performance of the MDC. The results obtained using the emission model and presented in Fig. 6 show that the MDC efficiency drops by ~ 11.5%. Including the secondary emission from the electrodes increases the simulation time by a factor of 5, and was therefore considered only in the final design. Further geometrical optimization should enhance the efficiency of the MDC. Based on the 20% interaction efficiency, as demonstrated in Fig. 4(b), and the 77.1% collection efficiency achieved for the 4-stage depressed collector with secondary electron emission, the overall efficiency of the 2D-PSL Cherenkov oscillator can be increased to 52.2%.

## Conclusions

The concept of efficient new surface wave sources with multi-stage depressed collectors has been presented and discussed. The demonstration of single-frequency, coherent emission in Cherenkov oscillators based on grossly oversized interaction cavities ${(\varvec{o}}_{\varvec{p}}>11)$ constitutes a major breakthrough in source development; particularly relevant to addressing the growing need for efficient, powerful mm-waves required to drive energy into dense fusion plasmas. Suitable initial parameters for the 2D-PSL interaction cavities were established using theoretical and analytical techniques, and thereafter numerically optimized to achieve high electronic efficiencies. It has been shown that, even for sources based on highly overmoded ${\varvec{o}}_{\varvec{p}}>11$ cavities, the surface field decays beyond the lattice boundary where it can effectively interact with the electron beam, demonstrating that the limit in ${\varvec{o}}_{\varvec{p}}$ has not been reached.

This is an important outcome, showing the potential to extend the spectral range of these devices well into the THz regime with even higher ${\varvec{o}}_{\varvec{p}}$. While the close proximity of the surface field to the metallic wall may impact on the transport of the electron beam, the potential to operate with higher order radial surface modes presents a viable solution. As expected, the transient time, from start-up to steady-state operation, has been shown to depend on the beam-wall separation. An exponential increase in the start-up time as a function of the separation has been simulated, showing good agreement with theoretical and experimental studies in beam grating coupling.

An exciting outcome of this work is the presentation of a novel energy recovery scheme demonstrating the ability to achieve competitive electronic and overall efficiencies as compared to existing source technology. Numerical PiC simulations show that the overall efficiency of the Cherenkov oscillators with ${\varvec{o}}_{\varvec{p}}\cong9$ can be increased using a multi-stage energy recovery system, with similar efficiency enhancement projected for sources with ${\varvec{o}}_{\varvec{p}}\cong11$and above. A comprehensive numerical study of multi-stage depressed collectors, with a varying number of stages, has been undertaken. Based on these studies, the best compromise between efficiency and complexity, was observed for a 4-stage depressed collector.

Through careful design and optimization, an increase in the overall efficiency of a ${\varvec{o}}_{\varvec{p}}\cong9$ Cherenkov oscillator from 20% (electronic efficiency) to 52.2% (68.7% if secondary emission is neglected) has been achieved. Secondary electron emission has been shown to be highly detrimental to the performance of the depressed collector. However, optimizing the collector geometry for secondary mitigation, by incorporating these secondary electrons into the full optimization routine, will increase the overall efficiency above 60%, with substantive impact potential in many important applications. Moreover, these novel design principles can be extended to other electron beam driven devices, further demonstrating the broad scope of this work.

Good agreement between theory and simulation has been observed for both the Cherenkov oscillator and the multi-stage depressed collector. Overall, the potential for Cherenkov oscillators based on vastly oversized interaction cavities with compatible multi-stage energy recovery systems is clear. These efficient, continuous-wave radiation sources operate within the desired 80–250 GHz range for plasma heating and current drive for fusion energy. The simulations demonstrate the potential of these 2D-PSL MDC sources to be competitive with established source technologies when operating within the power and frequency ranges required for fusion science and numerous other high-impact applications. Moreover, the scalability of both devices enables a promising route towards bridging the long-standing THz gap.

## Data Availability

Data underpinning this publication is available from the University of Strathclyde KnowledgeBase at Univ. Strathclyde, Glasgow, U.K., Tech. Rep., 2022, doi:10.15129/601bedb4-000e-430c-92f6-693afb06cce8.

## Abbreviations

2D PSL: Two-dimensional periodic surface lattice
MDC: Multi-stage depressed collector
CW: Continuous wave
STEP: Spherical tokamak for energy production
MW: Megawatt
